# Survey data on the Italian public's perception of the Russian invasion of Ukraine (March—August 2022)

**DOI:** 10.1016/j.dib.2024.110579

**Published:** 2024-05-31

**Authors:** Mario Angelelli, Cosima Valentino, Enrico Ciavolino, Mariano Longo, Sergio Salvatore, Pierpaolo Limone, Serena Arima, Terri Mannarini

**Affiliations:** a Department of Human & Social Sciences, University of Salento, via di Valesio, 73100 Lecce, Italy; b WSB Merito University in Gdansk, Poland; c UniPegaso Online University, Centro Direzionale Isola F2, 80132 Napoli, Italy

**Keywords:** Solidarity, Refugees, Threat, Emotions, Coping strategies, Trust, Future

## Abstract

This article presents data collected through Computer-Assisted Web Interviewing (CAWI), conducted in Italy with the aim of exploring the Italian population's reaction to the Russian invasion of Ukraine in February 2022 and monitoring this reaction for the first six months of the attack through a six-round survey. Each round involved a representative sample of approximately 1010 (1007–1015) Italian adult citizens. Participants were asked questions about emotional reactions to the Russian invasion, coping strategies used, solidarity behaviour toward Ukrainian refugees, perceptions of refugees as a threat to the country, trust in national and international authorities to manage the international situation, and prospects for the future. Demographic data on the respondents were also collected. The survey design was developed by a research group from two universities (the University of Salento and the University of Foggia) and a European research centre, EICAP (European Institute of Cultural Analysis for Policy). The data provided in this article is a resource for researchers, public authorities, and other parties interested in surveying and studying public opinion. This dataset can be used to explore a wide range of topics, including prosocial behaviour and attitudes towards refugees in humanitarian emergencies.

Specifications Table

**Table:** 

Subject	Social and Personality Psychology
Specific subject area	Attitudes and emotional responses to armed conflict and to the forcibly displaced
Type of data	Raw data with numbers and labels (.csv file) Codebook (.txt file) Text version of the survey questions, both in English and Italian (.pdf file)
Data collection	Data was collected in 6 rounds by a survey company in Italy, which recruited respondents by distributing email invitations to its panels. A representative population sample of Italian adults aged 18 and over was selected (respondents varied between 1007 and 1015 across rounds), randomly stratified by gender, age, and area of residence.
Data source location	Country: Italy
Data accessibility	Repository name: Mendeley data Data identification number: 10.17632/7sx9288bmx.2 Direct URL to data: https://data.mendeley.com/datasets/7sx9288bmx/2

## 1 Value of the Data

• The data provide valuable insights into public perceptions, reactions, and attitudes towards the armed conflict between Russia and Ukraine and towards Ukrainian refugees. It is of great importance to understand the views of Italian citizens on the conflict and the forcibly displaced, as well as its consequences. As such, the data are consistent with polls and surveys that monitor public opinion related to Russia's war against Ukraine both at the EU level and in the Member States (for a collection, see https://www.europarl.europa.eu/at-your-service/en/be-heard/eurobarometer/public-opinion-on-the-war-in-ukraine). The data could be of relevance for gathering similar data in other European countries and making comparisons between multiple European country-based representative samples.
• The data provide statistically significant sample sizes for national perceptions, particularly in terms of emotional reactions, coping strategies, solidarity behaviour toward refugees, perceptions of refugees as a threat to the country, trust in national and international authorities, and prospects for the future. In addition, by including multiple rounds of data collection, the data can be used to identify trends and patterns over a 6-month period.
• Researchers, social and public sector leaders, and policy-makers can benefit from this dataset by conducting further analyses of perceptions. Indeed, the data provide a valuable source of information on the links between individual responses to armed conflict and humanitarian emergencies and attitudes and assistance provided to refugees and asylum seekers and could therefore be of interest to social scientists with different backgrounds (i.e., primarily political scientists, social psychologists, and sociologists, but also social statisticians).
• While some forthcoming articles have used the data, focusing mainly on the role of emotions and perceived threats in refugee aid, these analyses have not been exhaustive.

## 2 Background

The primary goal of the survey was to explore and monitor public reaction to the Russian invasion of Ukraine as it unfolded, covering the first six months of the conflict, and secondarily to estimate population-level statistics on this topic using a nationally representative sample. In addition, a secondary goal was to make descriptive statistics and analyses of population averages available and accessible to the general public in real time via an open dashboard (https://dash-developer.shinyapps.io/temp-app/). Key concepts covered in the survey include individuals' responses to the humanitarian emergency created by the Russian attack on Ukraine, specifically the emotional response and strategies used to cope with such a disruptive event. The survey also includes the measurement of attitudes (toward the Ukrainian people fleeing their country, toward the institutions involved in resolving the conflicts, toward the personal and collective future) and a behavioral measure of aid provided to Ukrainian refugees since the Russian attack. Researchers will be able to use this dataset to perform a variety of statistical analyses.

## 3 Data Description

The files associated with this article include:

(1) Text version of survey questions, both in English and Italian, and response format (Questionnaire_EN_IT.pdf): The survey encompassed 41 questions, plus the demographics: gender, age, education, profession/employment, and area of residence (municipality, province, region, area).
(2) The raw survey data in .csv file format (Dataset Ukraine.csv): The survey data file contains 49 variables and data from each of the 1013 respondents. Blank entries in the dataset indicate that specific questions were not applicable to certain respondents.
(3) Text version of the codebook (CodeBook Ukraine.txt): The codebook provides explanations and details regarding all variables included in the survey data file.

The survey questions covered the following themes:

- Emotional response. Following theories with a discrete view of emotions (for a review, see [1]), respondents were asked to rate the intensity of the following 15 discrete emotions in relation to Ukraine's invasion on a 1 (= not at all) to 5 (= very much) point scale: sadness, anger, fear, disgust, contempt, joy, surprise, weariness, concern, anguish, guilt, shame, compassion, and hope. Indifference was added as a neutral affective state.
- Coping response. In relation to the invasion of Ukraine, respondents were asked to rate the frequency (response format from 1 = never to 5 =very often) with which they used both problem-focused strategies (actively doing something, two items) and emotion-focused strategies (efforts to change or reduce negative emotions, including distraction, two items) [2].
- Solidarity behaviour. Seven items were used to assess whether respondents had helped Ukrainian refugees in the past two weeks. Two types of support were considered, based on the distinction made by Thomas and McGarty [3,4] in the context of humanitarian emergencies: (a) Benevolent support (1. donated money to humanitarian organizations; 2. collected supplies for Ukrainian refugees; 3. Welcomed/received children or Ukrainian refugee families; 4. Helped bring refugees to Italy; 5. Joined a humanitarian organization as a volunteer to help Ukrainians on the ground); and (b) Activism (6. Signed a petition for peace; 7. Marched/demonstrated for peace). The response format was dichotomous (yes/no).
- Trust. Respondents were asked to rate, on a scale from 1 (= not at all) to 5 (= very much), how much they trust the following institutions in their ability to resolve the armed conflict in Ukraine: the United Nations, NATO, the European Union, the Italian Government, and the Catholic Church/the Vatican.
- Perspectives for the future. Respondents were asked to make predictions about the near future of their lives, the lives of their children, the country (Italy), and Europe. The response format was on a five-point scale, from 1 = much worse than now to 5 = much better than now (3 = same as now).
- Perceptions of refugees. Threat plays a key role in the perceptions and attitudes towards both voluntary and forced migrants as refugees and asylum seekers [[5], [6], [7], [8]]. Four items were developed to measure the perception of threats posed by the incoming Ukrainian refugees/asylum seekers in the country (Italy), rated on a scale from 1 (= not a problem) to 3 (= *a* major problem). Four types of threats were considered: concerns about financial burdens (economic threat), criminal acts (security threat), cultural differences (symbolic threat), and political consequences (political threat).

The demographic characteristics of the respondents, including age, gender, education, profession/employment, and area of residence, are displayed in Table 1.

**Table 1:** Overview of respondent distribution in terms of gender, age, geographical area, education, and profession/employment.

Variables	Category	N	%
Gender	M	2992	49.3
	F	3071	50.7
Geographical area	North-East	1164	19.2
	North-West	1615	26.6
	Center	1435	23.7
	South	1179	19.4
	Islands	670	11.1
Education	Primary (elementary school)	24	0.4
	Primary (middle school)	655	10.8
	Secondary	3516	58.0
	Tertiary	1868	30.8
Profession/Employment	Self-employed	633	10.4
	Employee	2080	46.6
	Employee as collaborator	138	2.3
	Unemployed	494	8.2
	Homemaker	612	10.2
	Student	324	5.3
	Retired	936	15.3
	Other	118	2.0
Age	18–35	1431	23.6
	35–50	1758	30
	50–65	1850	30.5
	over 65	1024	16.9

Descriptive statistics and analyses of population averages are available at https://dash-developer.shinyapps.io/temp-app/.

## 4 Experimental Design, Materials and Methods

A six-round survey (one round every 30 days) was conducted in Italy between March and August 2022. An Italian survey company (Teseo) conducted the survey online. For each round, respondents were recruited by the survey company through its panels, drawing a representative sample of participants in terms of gender, age, and geographic distribution, as specified by the research group. Respondents were randomly selected by selecting a number of potential respondents for each cell in the sampling plan that was deemed sufficient to reach the target number.

This number of potential respondents is calculated in each sampling cell as the “reciprocal of the historical redemption rate,” or the historical ratio of respondents to panelists contacted. The historical redemption level changes in each sample cell because survey participation is not constant according to the stratification variables (gender, age, and area of residence).

For each survey, potential respondents were contacted by e-mail, and the questionnaire was completed online. The average time required to complete the task was 9 min and 57 s. When registering for the panel, panelists were required to read and accept the privacy policy, in accordance with the GDPR. Surveys were conducted in a pseudonymized manner, i.e., by separating the respondent's ID from the personal information provided by the panelist during registration.

The response rate was 26.7 % (1013 respondents out of 3794 panelists contacted). Including 171 over-quota respondents (i.e., people who were available to respond to the survey but did so after the gender, age, and geographic sampling quotas were saturated), the response rate was 31.2% [(1013+171)/3794].

## Limitations

The data acquisition was confined to a six-month period in 2022. Additional investigations may be conducted by collecting data related to subsequent years or over an extended period.

A second limitation is the record of the respondents’ gender as a binary variable. Introducing a non-binary gender variable could improve the sampling planning and the analysis of associations between the respondents’ characteristics and their responses or perceptions.

## Ethics Statement

No ethical approval for this survey was required. All procedures conducted in the study adhered to the ethical standards set by the 1964 Helsinki Declaration and its subsequent amendments, or equivalent ethical standards, as well as the ethical codes of the American Psychological Association and of the Italian Psychology Association.

## CRediT authorship contribution statement

**Mario Angelelli:** Writing – review & editing, Software. **Cosima Valentino:** Writing – original draft. **Enrico Ciavolino:** Methodology, Software. **Mariano Longo:** Funding acquisition. **Sergio Salvatore:** Conceptualization, Funding acquisition. **Pierpaolo Limone:** Funding acquisition. **Serena Arima:** Writing – review & editing, Software. **Terri Mannarini:** Conceptualization, Supervision, Project administration, Investigation, Writing – review & editing.

## Data Availability

Italian public perception of the Russian invasion of Ukraine (Original data) (Mendeley Data).
